# Multicomponent analysis of the tumour microenvironment reveals low CD8 T cell number, low stromal caveolin-1 and high tenascin-C and their combination as significant prognostic markers in non-small cell lung cancer

**DOI:** 10.18632/oncotarget.18880

**Published:** 2017-06-29

**Authors:** David Onion, Mark Isherwood, Naveen Shridhar, Mikalena Xenophontos, Madeleine L. Craze, Laura J. Day, María A. García-Márquez, Robert G. Pineda, Alexander M. Reece-Smith, John H. Saunders, John P. Duffy, Richard H. Argent, Anna M. Grabowska

**Affiliations:** ^1^ *Ex Vivo* Cancer Pharmacology Centre of Excellence, Cancer Biology, Division of Cancer and Stem Cells, School of Medicine, University of Nottingham, Nottingham, UK; ^2^ University of Nottingham Flow Cytometry Facility, School of Life Sciences, University of Nottingham, Nottingham, UK; ^3^ Department of Thoracic Surgery, Nottingham University NHS Trust, City Hospital Campus, Nottingham, UK

**Keywords:** non-small cell lung cancer, tumour microenvironment, caveolin-1, tenascin-C, T cell

## Abstract

The complex interplay of the tumour microenvironment (TME) and its role in disease progression and response to therapy is poorly understood. The majority of studies to date focus on individual components or molecules within the TME and so lack the power correlative analysis. Here we have performed a multi-parameter analysis of the TME in 62 resectable non-small cell lung cancer (NSCLC) specimens detailing number and location of immune infiltrate, assessing markers of cancer-associated fibroblasts, caveolin-1 and tenascin-C, and correlating with clinicopathological details, as well as markers of disease progression such as epithelial-to-mesenchymal transition (EMT). The influence of individual parameters on overall survival was determined in univariate and multivariate analysis and the combination of risk factors and interplay between components analysed. Low numbers of CD8 T cells, low stromal levels of caveolin-1 or high levels of tenascin-C were significant prognostic markers of decreased overall survival in both univariate and multivariate analysis. Patients with two or more risk factors had dramatically reduced overall survival and those with all three a median survival of just 7.5 months. In addition, low levels of tumour E-cadherin correlated with reduced immune infiltrate into the tumour nests, possibly linking EMT to the avoidance of CD8 T cell control. The multicomponent approach has allowed identification of the dominant influences on overall survival, and exploration of the interplay between different components of the TME in NSCLC.

## INTRODUCTION

The influence of the tumour microenvironment (TME) upon non-small cell lung cancer (NSCLC) remains poorly defined with conflicting reports in the literature surrounding the influence of immune components as well as cancer-associated fibroblasts (CAFs) on disease progression and outcome. The radical success of the early trials of PD1/PD-L1 checkpoint inhibitor immunotherapies appears to demonstrate that the TME has a guiding influence on NSCLC disease outcome and points the way for further exploration of TME-targeted therapies [1].

The complexity of the TME, comprising fibroblasts, endothelial cells, pericytes, immune infiltrate and other cells along with their secretions and extracellular matrix, make it a challenging system to study and many reports focus on one part in isolation, potentially overlooking important interactions. The major non-tumour cell type found in the NSCLC microenvironment are fibroblasts, which surround and penetrate through the tumour in a web-like pattern. This produces an architecture common to many solid tumours where groups of tumour cells are clustered in so called nests (or islets) and separated from adjacent nests by a swathe of fibroblasts, often including the vasculature. The origin and even definition of CAFs remains controversial [2] and indeed CAFs themselves are a heterogeneous and dynamic population that exists in co-development with the tumour [3].

Biomarkers of CAFs are beginning to show promise in the prognosis of a number of solid tumours but have been most extensively studied in breast cancer. Caveolin-1, when expressed by fibroblasts, appears to negatively regulate tumour cell growth and the loss of expression is strongly correlated with reduced overall survival, tamoxifen resistance and metastasis in breast cancer [4, 5] and high Gleason score in prostate cancer [6]. Cav-1 deficient fibroblasts upregulate glycolytic enzymes and secrete energy rich metabolites which can be used by the proliferating tumour cell in the so called ‘reverse Warburg effect’ [7]. Tenascin-C expression by fibroblasts has been associated with disease in a number of cancers including bladder, brain and colon but only definitively linked with poor prognosis or survival in breast and lung [8, 9]. Tenascin-C appears to be involved in the metastatic process and is often found at the tumours leading edge [10], it plays a role in epithelial-to-mesenchymal transition (EMT) [11] and has also been shown to block T cell activation [12]. As CAFs are known to modulate a number of treatments, CAF markers may aid patient stratification and chemotherapy choice and may also provide novel targets for future chemotherapeutics.

Immune infiltrate is commonly observed in NSCLC and is chiefly composed of T cells and macrophages. There have been conflicting reports on the importance of the composition and influence of location on disease progression. Al-Shibli et al. [13] found a significant association between increased stromal and intratumoural CD8+ T cells and improved disease-specific survival whereas Wakabayashi et al. found an association between increased intratumoural CD8+ T cells and shorter overall survival [14]. Hiraoka et al. found that concurrent CD4+ and CD8+ T cell infiltration is a favourable prognostic factor [15]. More recently, Djenidi et al. reported that CD8+CD103+ tumour infiltrating T cells are a prognostic factor for survival in NSCLC [16] and the success of checkpoint immunotherapy trials indicates that T cells can play an important role in disease control [1]. T-regulatory cells are implicated in the dampening of anti-tumour immune responses in a number or tumour types [17] have been reported to influence disease progression in NSCLC. An increased number of total T-reg cells and a higher proportion of total T-reg cells relative to tumour-infiltrating lymphocytes (TILs) has been associated with worse recurrence-free survival [18]. A study by Tao et al. also found an association between increased total T-reg levels and poor overall and relapse-free survival [19]. Approximately one third of the immune infiltrate in NSCLC are tumour-associated macrophages (TAMs) [20], the majority of which are alternatively activated M2 macrophages [21]. M2 macrophages are implicated in disease progression in a number of tumour indications and can both act to supress the anti-tumour immune response but also facilitate tumour growth and metastasis.

In order to better understand the influence of the TME we sought to profile the major cellular TME components namely, fibroblasts, T cells (CD3, CD8 and T-reg) and macrophages and relate these to patient survival in univariate and multivariate analysis in the context of the known clinicopathological data.

## RESULTS

### Immune infiltrate

**Figure 1:**
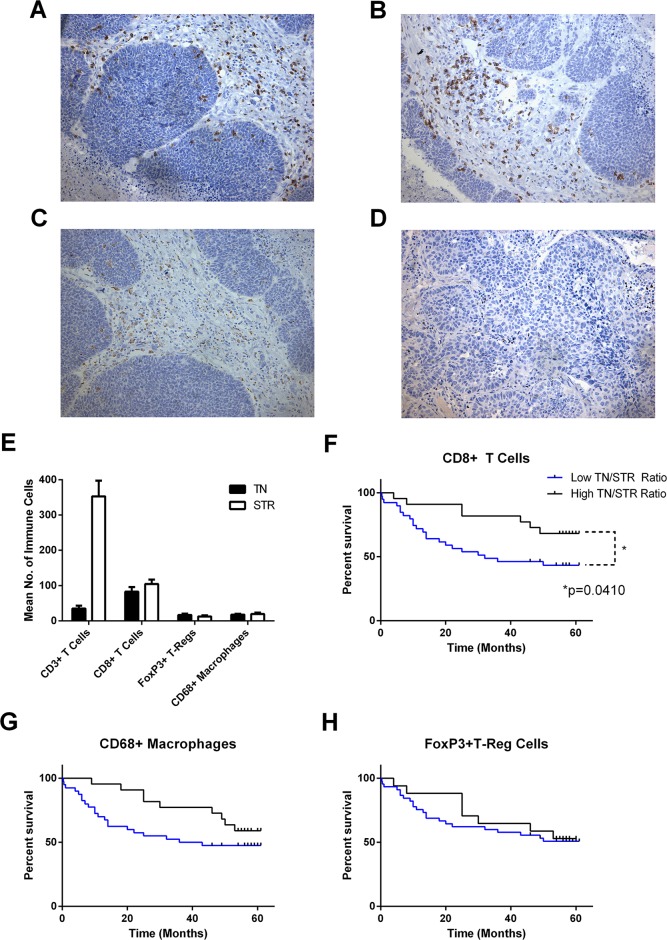
Number, localisation and influence of immune infiltrate on overall survival in NSCLC Serial sections of FFPE NSCLC tissue (n = 62) were immunohistochemically stained for CD3, CD8, CD68 and FoxP3. Cells were enumerated and location categorised as tumour nest (TN), stromal (STR) and the resultant ratio (TN/STR). Example images of staining for **(A)**, CD3; **(B)**, CD8; **(C)**, CD68; **(D)**, FoxP3. **(E)**, mean number of tumour-associated cells in each location; error bars represent standard errors of the mean. **(F, G, H)** Kaplan-Meier plots of overall survival of patients divided by low and high (below or above the mean) TN/STR ratio of **(F)**, CD8 cells, **(G)**, CD68+ Macrophages and **(H)**, FoxP3+ T-regulatory cells. Significance <0.05 as measured by Log-rank (Mantel-Cox) test are shown.

A cohort of 62 tumour specimens from patients with operable NSCLC (stage 1-3) were analysed for their immune infiltrate by immunohistochemical analysis. The number of each cell-type and location in tumour nest or stroma was determined for total T cell (CD3), CD8 T cells (CD8), T-reg (FoxP3) and macrophages (CD68) and related to survival by Kaplan-Meier analysis (Figure 1). In line with other reports the samples contained high numbers of CD3+ T cells predominantly in the tumour stroma; approximately two-thirds of these were CD8+ T cells which were evenly distributed between tumour nest and stroma. A smaller number of CD68+ macrophages and FoxP3+ T–regs were observed with roughly equal distributions between tumour nest and stroma. Number and distribution of immune cells is shown in Figure 1E. Kaplan- Meier survival curves were generated for all markers dividing the data between high (above mean) and low (below mean) infiltration into either tumour nest, tumour stroma or the ratio between the two. The strongest trends were observed by examining the tumour nest to stromal ratios (TN/STR) where those with higher TN/STR ratios of CD8, CD68 or FoxP3 had higher overall survival (Figure 1F-1H); however, this only reached significance for CD8+ T cells (p=0.041 Mantel-Cox Log-rank test, p=0.0294 Gehan-Breslow Wilcoxon test).

### Cancer- associated fibroblasts

**Figure 2:**
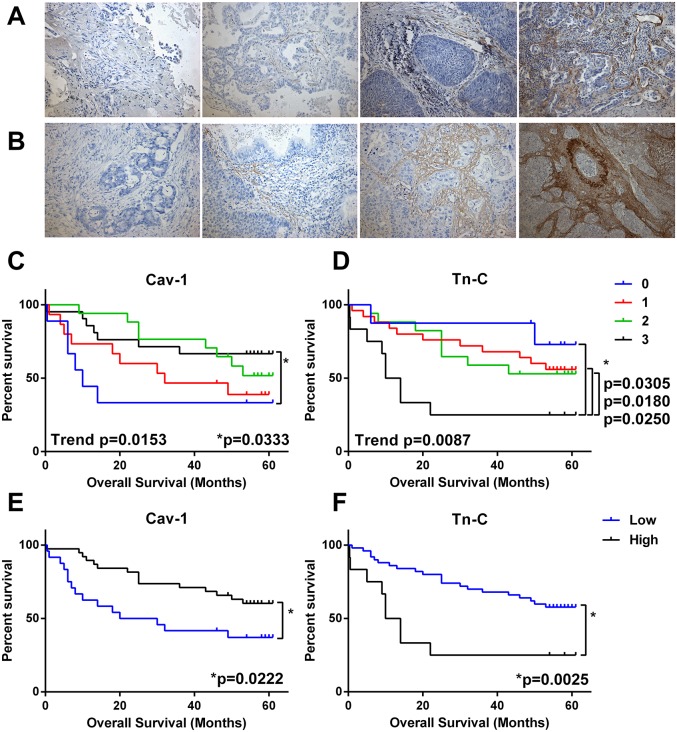
Predictive value CAF markers in NSCLC Serial sections of FFPE NSCLC tissue (n = 62) were immunohistochemically stained for caveolin-1 (Cav-1) or tenascin-C (Tn-C). Stromal-associated staining was categorised as absent (0) or low-high (1-3) and compared to overall survival in Kaplan-Meier survival analysis. **(A)** Example images of Cav-1 scoring 0-3 (left to right). **(B)** Example images of Tn-C scoring 0-3 (left to right). **(C)** Survival by level of Cav-1 (0-3 score). **(D)** Survival by level of Tn-C (0-3 score). **(E)** Survival by level of Cav-1 grouped as low (0-1 score) or high (1-2 score). **(F)** Survival by level of Tn-C grouped as low (0-2 score) or high (3 score). ^*^ = significance P <0.05 as measured by Log-rank (Mantel-Cox) between groups and trend= significance of Log-rank test for trend.

The cohort was examined for two CAF-associated proteins with putative prognostic significance: tenascin-C, which has been shown to be up-regulated in a number cancers, and caveolin-1, the loss of which is a strong predictor of overall survival in breast cancer and is a promising marker in NSCLC [5, 8, 22]. Degree of immunohistochemical staining in the stromal cells only was graded 0-3 (0 = absence of stain, 1-3 low to high staining) and survival of groupings analysed by Kaplan-Meier plots (Figure 2). Decreased Cav-1 staining in stromal fibroblasts was significantly associated with decreased overall survival both as a trend (Log-rank test for trend p=0.0153) or when comparing low (0-1 score) to high (2-3 score) (Log-rank test p=0.0222) in NSCLC. Overall survival was significantly reduced in patients with increased tenascin-C (Log-rank test for trend p=0.087) and there was a marked difference in survival between patients with the highest amount of tenascin-C (3 score) and those with other scores (0-2) or when low staining (0-2) was compared to those with high staining (Log rank test p=0.0025). While overall there was a modest trend to inverse relationship between Tn-C and Cav-1 levels no significant relationship existed (Supplementary Figure 1) suggesting these are two distinct biological processes.

### Epithelial-to-mesenchymal transition

**Figure 3:**
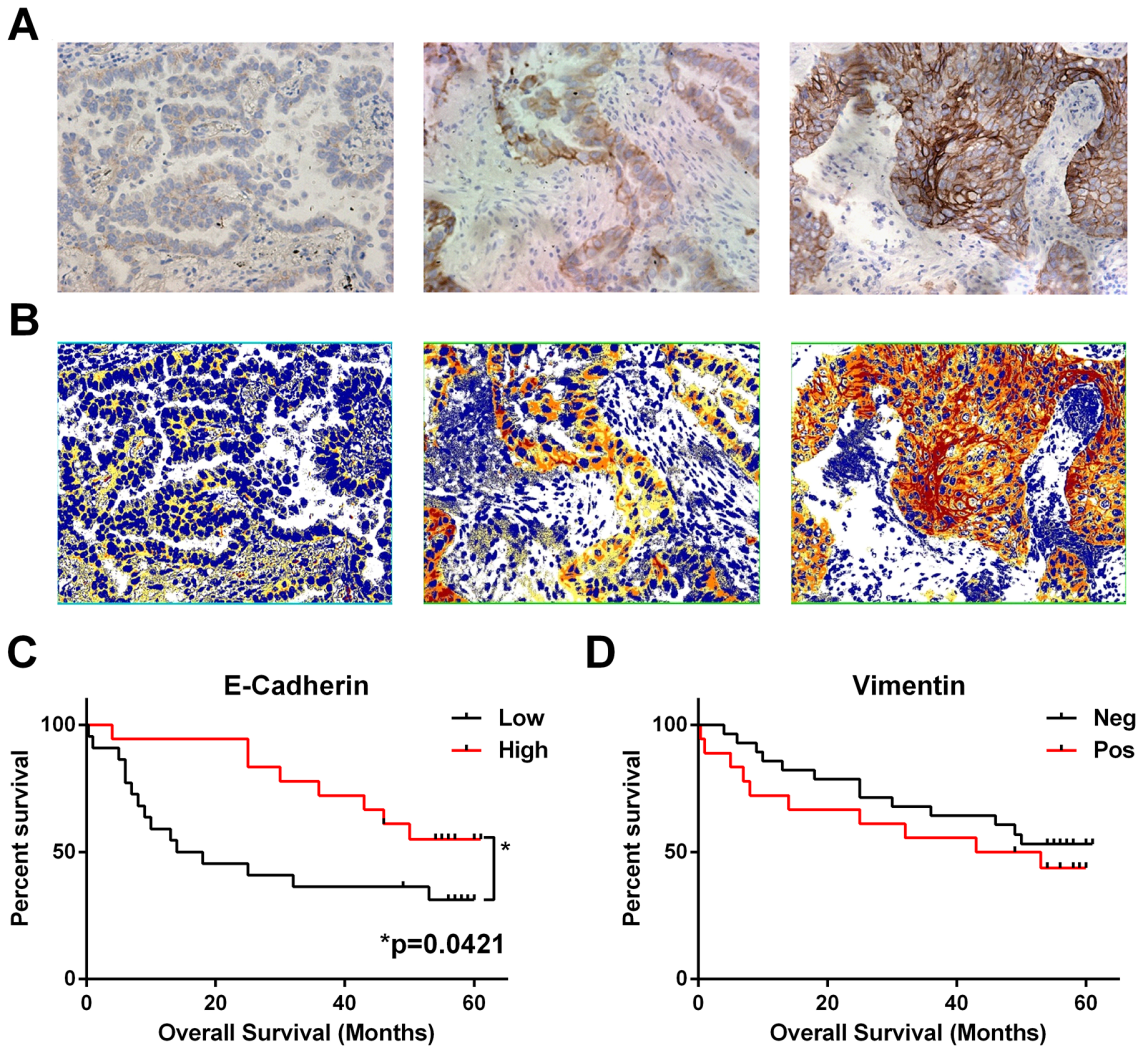
Loss of epithelial marker E-cadherin correlates with worse overall survival Serial sections of FFPE NSCLC tissues (n = 41) were immunohistochemically stained for E-cadherin or vimentin. **(A)** Example images of week, moderate and intense E-Cadherin staining (left to right). **(B)** Matched image analysis (to A) produced by Image Scope Positive Pixel Algorithm with pixels coloured as weak (yellow), moderate (orange) or intense (red), extracted values were used to calculate a mean H-Score for each patient. **(C)** Patient survival based on E-Cadherin H-Score grouped as low or high (below or above) the mean H-Score. **(D)** Presence of vimentin staining in any of the tumour nests was categorised as positive, and total absence as negative. ^*^ = significance P <0.05 as measured by Log-rank (Mantel-Cox) between groups.

In order to look for evidence of EMT in the NSCLC specimens we quantified E-cadherin levels in the tumour cells by H-Score analysis and also scored them for expression of the mesenchymal marker vimentin. Figure 3 shows that low E-cadherin (below mean H-Score: 55% of patients) was predictive for worse overall survival (Log-rank test p=0.0421, Gehan-Breslow-Wilcoxon test p=0.0182) whereas the presence of any vimentin staining in any of the tumour cells was not predictive on its own or in combination with E-cadherin levels.

**Figure 4:**
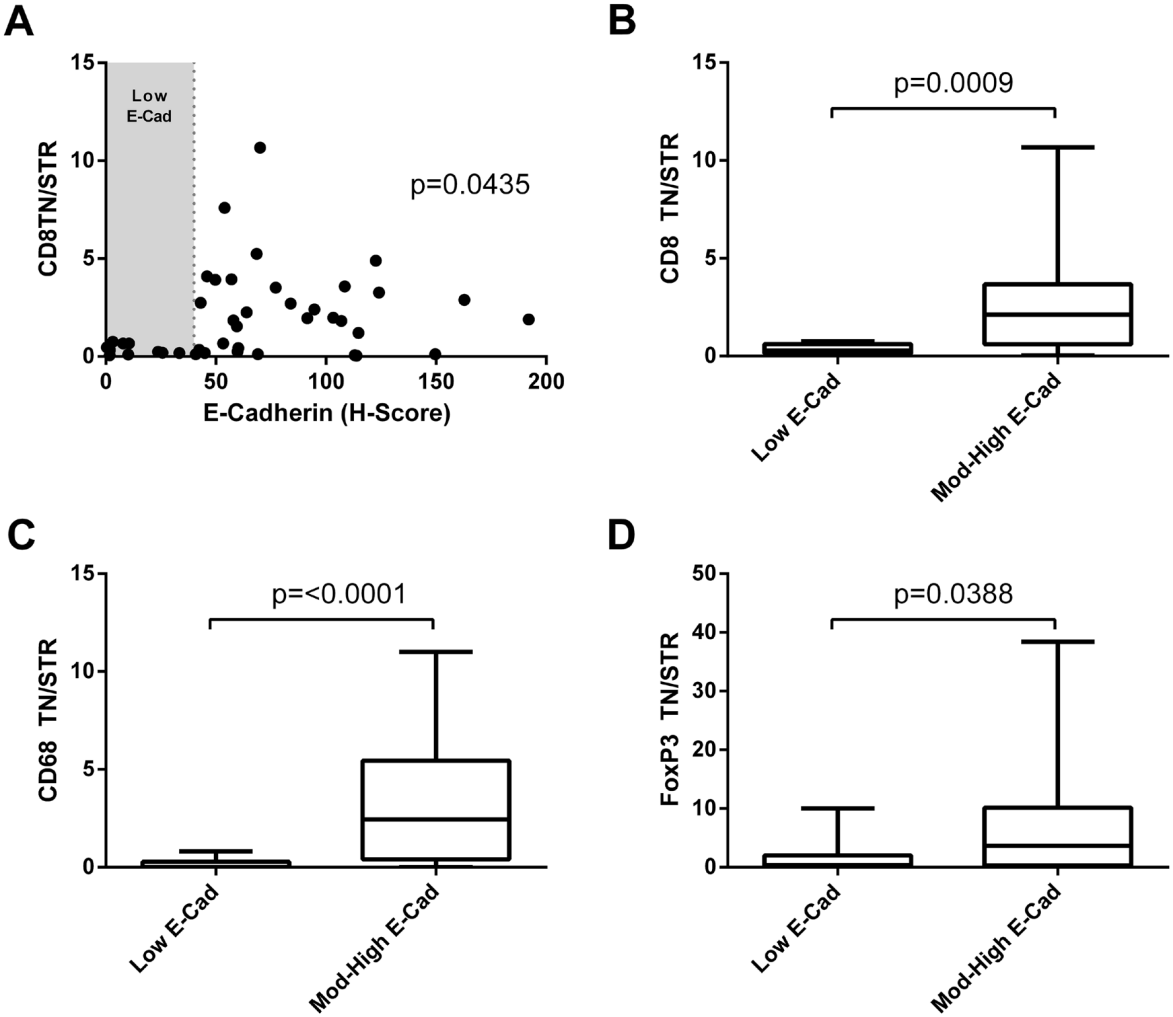
Immune infiltration to the tumour nests correlates with low E-cadherin level **(A)** Correlation of E-cadherin H-score and CD8 tumour nest/stromal ratio. Significance of Spearman's non-parametric correlation is shown. **(B, C, D)** TN/STR ratio of immune infiltrate in tumours with low E-cadherin (low H-score shown as shaded area in figure A), compared with those with moderate or high E-cadherin for: **(B)** CD8 T cells; **(C)** CD68+ macrophages or **(D)** FoxP3+ T-regulatory cells. Significance of Mann-Whitney unpaired non-parametric T- test are shown.

It has been recently demonstrated that CD8 T cell expression of CD103 is required for retention in tumour nests and it is these cells that possess tumour lytic ability [16]. As the receptor for CD103 is E-cadherin we investigated if lymphocyte infiltrate into the tumour nest correlated with E-cadherin expression (Figure 4A). E-cadherin levels showed a correlation with high CD8 TN/STR (rank Spearman p=0.0435) and from this it was noted that those cells with low levels of E-cadherin had significantly lower numbers of CD8, FoxP3, and CD68 cells in the tumour nest but not in the stroma (Supplementary Figure 2) and hence lower TN/STR ratios (Figure 4B–4D).

### Multivariate analysis and combinatorial risk

While the univariate Kaplan-Meier and Log-rank tests had identified low CD8 TN/STR, high Tn-C, low Cav-1 and low E-cad as predictors of worse overall survival, it was also important to take into account the variation in clinical features to determine whether they are truly independent predictors of outcome. A multivariate analysis (Cox Regression) was performed including covariates age, gender, stage of disease and disease sub-type. Results of multivariate analysis for our investigational markers are shown in Table 1, where it can be seen that CD8 numbers (total, tumour nest or stromal number) was a significant predictor of survival with small hazard ratios; however, high CD8 TN/STR ratio was a significant predictor of better overall survival (p=0.037) with hazard ratio of 0.645 (95%CI 0.427-0.974). Both CAF markers were predictive of outcome with high levels of Tn-C predictive of worse overall survival (p=0.005, HR 2.309) and high levels of Cav-1 predictive of increased survival (p=0.038, HR 0.635). Although included in the analysis age, gender, stage of disease and disease sub-type were not independent predictors of overall survival in this early stage disease cohort.

**Table 1:** Association of TME markers with overall survival in multivariate analysis

	No. of Patients	No. of Deaths	HR (95%CI)	P Value
***Total***				
CD8	62	30	1.091 (1.006-1.185)	**0.036**
CD68	62	30	1.007 (0.983-1.031)	0.580
FoxP3	62	30	0.955 (0.970-1.021)	0.728
***Tumour Nest***				
CD8	62	30	0.917 (0.842-0.998)	**0.044**
CD68	62	30	0.981 (0.940-1.025)	0.396
FoxP3	62	30	1.024 (0.977-1.074)	0.319
***Stromal***				
CD8	62	30	0.913 (0.842-0.991)	**0.029**
CD68	62	30	1.005 (0.982-1.029)	0.654
FoxP3	62	30	1.001 (0.977-1.026)	0.951
***TN/STR***				
CD8	62	30	0.645 (0.427-0.974)	**0.037**
CD68	62	30	0.889 (0.745-1.061)	0.192
FoxP3	62	30	0.920 (0.823-1.028)	0.140
***CAF Markers***				
Cav-1	62	30	0.635 (0.413-0.635)	**0.038**
Tn-C	62	30	2.309 (1.287-4.143)	**0.005**
***EMT Markers***				
E-Cadherin	41	22	1.642 (0.436-6.183)	0.464
Vimentin	41	22	1.150 (0.180-7.329)	0.883
***Combination***				
CD8 TN/STR & High Cav-1 & Low Tn-C	62	30	3.207 (1.907-5.392)	**<0.001**

Bold type indicates significance p<0.05. TN/STR= tumour nest: stromal ratio.

**Figure 5:**
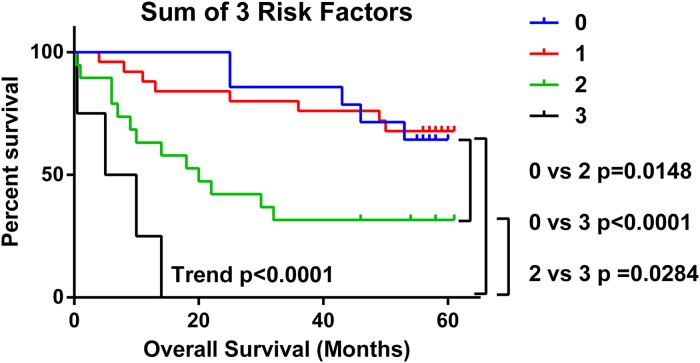
Presence of multiple TME risk factors strongly predicts for poorer overall survival The number of risk factors (low CD8 TN/STR, high Tn-C and low Cav-1) present in NSCLC samples from each patient was calculated and analysed by in Kaplan-Meier survival analysis. Significance <0.05 as measured by Log-rank (Mantel-Cox) between groups is shown. Trend= level of significance of Log-rank test for trend.

We determined the total number of the three independent risk factors (Tn-C high, Cav-1 low and CD8 TN/STR low) that each patient had and plotted this, sum of total risk factor against overall survival (Figure 5). Total risk factor score was highly predictive of worse overall survival as a trend (p<0001) and when comparing 0 risk factors to 2 (p=0.0148) or 3 risk factors present (p<0.0001). In a multivariate analysis with age, gender, stage of disease and disease sub-type as covariates the total risk factor score was a significant predictor of worse overall survival p<0.0001, HR 3.207 (95%CI 1.907-5.392) (Table 1).

## DISCUSSION

Using a multi-parameter approach to profile components of the TME we have been able to identify low CD8 number and absence of infiltration into the tumour nests as well as the over-production of Tn-C or reduced production of Cav-1 by CAFs as significant predictors of shortened overall survival in NSCLC. Uniquely by measuring all the parameters together we were able to determine that low levels of tumour E-cadherin, which appeared predictive of worse overall survival in univariate but not multivariate analysis, was a confounder of immune infiltration: presence of E-cadherin at moderate or high level was critical for the presence of CD8+ T cell, FoxP3+ T-regulatory cells and CD68+ macrophages in the tumour nests. While we only analysed two indicative markers here (E-cadherin and vimentin) any shift to a mesenchymal phenotype caused by EMT would cause loss of E-Cadherin and may directly inhibit the CD8+ cytotoxic T cell control of cancer. The value of the multi-parameter approach was demonstrated by the strong predictive value of reduced overall survival for patients that had more than one of the three risk factors (low CD8, high Tn-C or low Cav-1), with those with all three risk factors present having a median survival of just 7.5 months.

Whilst highly predictive of survival in breast cancer, low stromal Cav-1 has only recently been shown to be of prognostic significance in NSCLC. In a phase II trial of *nab*-paclitaxel and carboplatin in advance squamous NSCLC high stromal Cav-1 correlated with improved response rate and increased survival [22]. The analysis is complicated in NSCLC by tumour cell upregulated Cav-1 being reported to correlate with poorer survival and resistance to therapy [23, 24]. Our study for the first time demonstrates across the pathological subtypes that low stromal Cav-1 is associated with worse overall survival in operable NSCLC patients. Stromal Cav-1 expression in breast and ovarian cancer is driven in concert with tumour cell oncogene expression with the resulting oxidative stress causing a switch from normal to CAF-like phenotype. The activated fibroblasts are thought to produce energy rich metabolites that help support the growing tumour via the reverse Warburg effect [25]. This metabolic coupling between the TME and tumour cells represents an as yet untapped target for novel chemotherapeutics which are desperately needed for the treatment of NSCLC.

Tenascin-C, a component of the extracellular matrix, is rarely seen in adult tissue other than in the bone marrow or lymphoid tissue or in cases of wound healing and solid tumours. While there is evidence in a number of tumour indications that larger isoforms are more predominantly expressed in tumour stroma there are few definitive reports correlating stromal Tn-C with disease progression or survival [9]. Tn-C expression was predictive of worse overall survival in a study of women with node negative breast cancer [26] and, in NSCLC, increased Tn-C has been shown to correlate with disease recurrence and inhibition of the effector function of TILs [12]. Here, in a cohort of operable NSCLC patients we see an association of Tn-C and overall survival in both univariate and multivariate analysis. Tn-C is expressed in primary and secondary lymphoid organs and has been shown to interact with and modulate T cell function and hence we looked for an association between immune cell number and localisation with the level of Tn-C, but none was found (Spearman's correlation P>0.05 for all combinations). Given Tn-C expression is usually localised to the tumour stroma and has been shown to supress TILs from NSCLC, effector T cells that localise to the tumour stroma may be inactivated by association with Tn-C and in part may explain why T cell localisation appears to be important in disease progression.

In this study we focussed on two proteins differentially expressed in cancer associated fibroblasts of other tumour indications but which have only very recently begun to be investigated in NSCLC. The results presented here indicate that a systematic investigation into role of cancer associated fibroblasts in the disease progression of NSCLC is warranted and may yield useful targets for future therapy.

Cancer is often referred to as the wound that never heals because of the ever-present inflammation and similarities to the wound healing process [27]. The progression to a more aggressive and invasive phenotype including that of EMT has more recently been linked with the inflammatory process at cellular and molecular levels [28]. Inflammatory cells such as TAMs are recruited to the tumour stromal interface and along with CAFs secrete cytokines including TNF-α and TGF-β to control the inflammatory response and to activate wound healing programmes. This signalling and the secretion of metalloproteinases by CAFs causes the activation of NF-κB in tumours leading to the activation of Snail, TWIST and Zeb and ultimately EMT [29–32]. These and other exogenous micro-environmental factors such as hypoxia and oxidative stress lead to a positive feedback loop further promoting EMT [33, 34] and increasing the inflammatory signals with consequences including immunosuppression mediated by T-regulatory cells and reduced efficacy of dendritic cells and cytotoxic T cells [35]. While at the molecular level, and usually in model systems, there are strong links between EMT and the TME, these are often difficult to study in human tumours. Localisation of immune cells has been reported as a predictor of progression [13–15] and the more recent inhibition of immune checkpoints suggest paracrine inhibition of cytotoxic T cells is critical for tumour progression [1]. Interestingly Djenidi et al. recently reported that the presence of CD8+ T cells expressing CD103 correlated with better overall survival in NSCLC, irrespective or localisation [16]. Induction of CD103 expression requires MHC-peptide/TCR engagement in the presence of TGF-β1 and hence *in situ* by engaging tumour cells in the context of TGF-β1 rich microenvironment [36]. The acquisition of this resident memory T cell phenotype (T_RM_) also includes up-regulation of checkpoint proteins including PD-1, rationalising the efficacy of PD-1 inhibitor trials and supported by *in vitro* tumour cell killing by CD8+ CD103+ cells when under PD-1 blockade [16]. The main receptor for CD103 is E-cadherin, the down-regulation of which is a key event in EMT. Here, we show for the first time that a low level of tumour cell E-cadherin is associated with low levels of immune cells (CD8+ T cells, CD68+ macrophages or FoxP3+ T-regs) in the tumour nest. This may be explained if E-cadherin/CD103 interaction is required to retain the cells in the tumour nest, after the initial TCR-peptide/MHC interaction in the presence of TGF-β1, in the case of CD8+ T cells. Subsequent migration to the stroma would potentially result in loss of CD103+ once TCR-peptide/MHC interactions are lost and cells would potentially interact with Tn-C further leading to their inactivation. Therefore, low E-cadherin, low tumour nest CD8 and low total CD103+ T cells would be indicative of tumours progressing though EMT with lack of T-cell control all ultimately as a results of microenvironment driven factors including CAF-derived TGF-β and oxidative stress.

By simultaneously profiling the intertwined components of the TME, we have identified individual factors that predict overall survival, a connection between immune retention and E-cadherin and ultimately combinatorial risk factors which identify those with dramatically reduced overall survival.

## MATERIALS AND METHODS

### Patients

Fresh surgical material from tumour resections at Nottingham University Hospitals NHS Trust, were collected with informed patient consent and National Research Ethics Service (NRES) approval (NRES REC 10/H0405/6) between March 2010 and April 2012. Samples were used in accordance with NRES approval (NRES REC 08/H0403/37). Patients were followed up to April 2015. Histological subtype and tumour stage were determined by pathologists at Nottingham University Hospitals NHS Trust according to Word Health Organisation criteria [37] and tumour, node, metastasis (TNM) stage criteria published by the International Association for the Study of Lung Cancer committee [38].

### Immunohistochemistry

Serial 5μm thick sections of formalin-fixed and paraffin-embedded (FFPE) tissue were cut for immunohistochemical analysis by microtome and mounted on polylysine-coated slides. Following deparaffinisation and re-hydration antigen retrieval was performed by heating to 98°C in a temperature controlled microwave in retrieval buffer according to manufacturer's instruction. Sections were then quenched, blocked in a 3% hydrogen peroxide solution for 10 mins, washed (H_2_0) blocked with streptavidin and then biotin (Vector Laboratories). Sections were subsequently washed in buffer, blocked in 5% rabbit serum and then incubated with the primary antibody or isotype and concentration matched negative control. After washing, sections were incubated with a secondary biotinylated rabbit anti-mouse antibody (DAKO), washed and incubated in avidin and then biotin complex (ABC) solutions according to manufacturer's instructions (Vector Laboratories). Sections were washed incubated with 3,3’-diaminobenzidine (DAB) (DAKO), washed and counterstained with haematoxylin. Subsequently the sections were washed, dehydrated and cleared before mounting a coverslip with Distyrene Plasticizer Xylene (DPX).

Antibodies used in this study were: mouse anti-human CD3 (DAKO, M7254, Clone F7.2.38) 7 μg/ml, mouse anti-human CD8 (DAKO, M710301, Clone C8/144B) 3 μg/ml, mouse anti-human FOXP3 (eBiosciences, 14-4777-82, clone 236A/E7) 2.5 μg/ml, mouse anti-human CD68 (DAKO, M0876, clone PG-M1) 0.02 μg/ml, mouse anti-human vimentin (DAKO, M0725, clone V9) 4 μg/ml, mouse anti-human E-cadherin (DAKO, M3612, clone NCH38) 3 μg/ml, mouse anti-human caveolin-1 (BD Biosciences, 610407, Clone 2297) 0.5 μg/ml and mouse anti-human tenascin-C (AbCam, Ab86182, Clone DB7) 5 μg/ml.

### Scoring and image analysis

#### Immune cell infiltrate

A total of six images for each tissue section were obtained at 20x magnification (18.2 mm^2^ field of view) using a Leica DFC480 colour-inverted microscope; with three tumour nest (TN) hotspot and three stromal (STR) hotspot images taken. The hotspots were defined as a field of view with the most apparent accumulation of stained cells in either areas of tumour epithelia or stromal cells (as determined by tissue architecture). The stained cells were subsequently counted using ImageJ version 1.46r, “cell counter” plug-in and a TN and STR total was obtained for each image. The sum of the TN and STR cell numbers from all 6 images was determined to allow the total ratio (TN/STR) to be calculated for a given tissue section.

### E-cadherin

On six fields of view E-cadherin was quantified using ImageScope software (Version 11.1.2.760) with ‘Positive Pixel Count v9.1’ algorithm and thresholds set at high positive- 200, positive- 150, weak positive- 100. The algorithm generated an H-score using the formula: H-Score = (3 x percentage of high positive pixels) + (2 x percentage of medium positive pixels) + (1 x percentage of low positive pixels).

### Vimentin

Slides were visually scanned by two blinded operators for any vimentin staining in any tumour cells and presence of any vimentin in tumour was scored as positive.

### Caveolin-1 and tenascin-C

Sections were scored by three blinded assessors for the degree of stromal staining 0= 0-1%, 1= 2-10%, 2= 11-50%, 3=>50%. Where scores from each assessor varied the majority (2 of 3 scorers) was recorded.

### Statistical analysis

Kaplan-Meier survival analysis was performed in GraphPad Prism software (v6.04) and Log-rank (Mantel-Cox) test P value reported. Where appropriate the Gehan-Breslow-Wilcoxon test P value was reported which is weighted for early death. Where there were more than two groups log-ranks test for trend was also reported.

For multivariate Cox-regression analysis data was analysed in SPSS Statistics (v22, IBM) with gender, age, histological type and stage as covariates alongside data from immunohistochemical staining.

Analysis of correlation between E-cadherin H-score and CD8 TN/STR ratio was performed in GraphPad Prism software using Spearman's non-parametric correlation. Difference in TIL TN/STR ratio or number of those patients with low E-cadherin H-Score and those with moderate or high E-cadherin was tested by Mann-Whitney unpaired non-parametric T-test (GraphPad Prism).

## SUPPLEMENTARY MATERIALS FIGURES


