# Supplementary material for: Multicomponent analysis of the tumour microenvironment reveals low CD8 T cell number, low stromal caveolin-1 and high tenascin-C and their combination as significant prognostic markers in non-small cell lung cancer
Source: Oncotarget. 2017 Jun 29;9(2):1760–71. doi: 10.18632/oncotarget.18880 (PMC5788597; doi:10.18632/oncotarget.18880)
Supplement: Supplementary file 1 [file oncotarget-09-1760-s001.pdf]

Multicomponent analysis of the tumour microenvironment reveals low CD8 T cell number, low stromal caveolin-1 and high tenascin-C and their combination as significant prognostic markers in non-small cell lung cancer

SUPPLEMENTARY MATERIALS

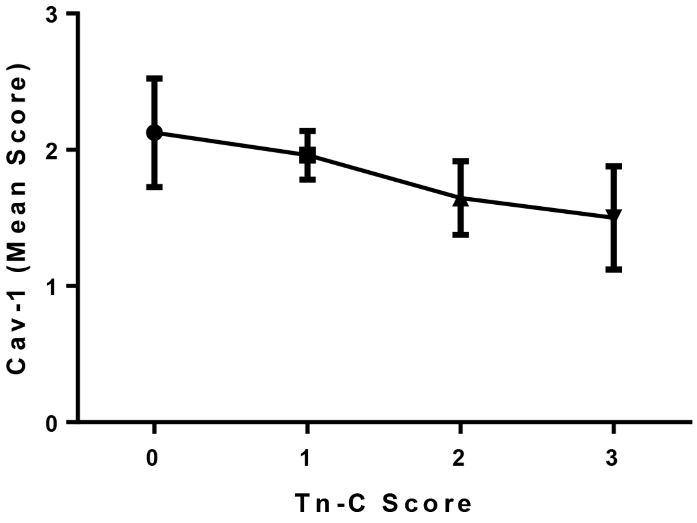

Supplementary Figure 1: Inverse relationship between Cav-1 and Tn-C in the tumour stroma. Samples were stained for Cav-1 and Tn-C as described in the main Materials and Methods. Sections were scored by three blinded assessors for the degree of stromal staining 0= 0-1%, 1= 2-10%, 2= 11-50%, 3=>50%. Error bars represent standard errors of the mean.

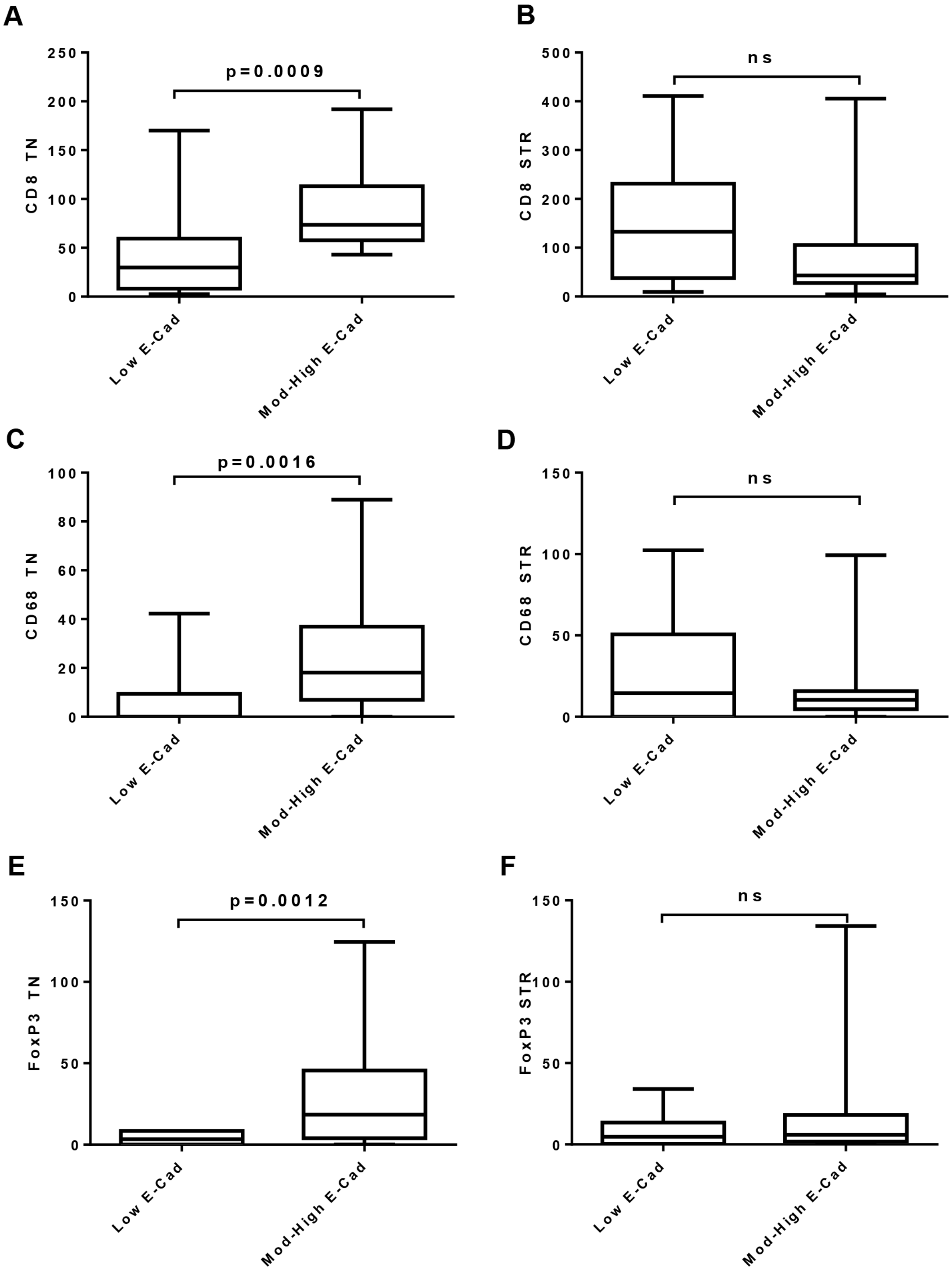

Supplementary Figure 2: Frequency of immune infiltrate in the tumour nest but not the tumour stroma correlates with expression of E-Cadherin on tumour cells. Frequency of immune infiltrate in tumours with low E-cadherin (H-Score) compared with those with moderate or high E-cadherin (medium or high H-Score) for: **(A & B)**, CD8 T cells; **(C & D)**, CD68+ macrophages; **(D & E)**, FoxP3+ T-regulatory cells in tumour nest (TN) **(A, C, E)** or stroma (STR) **(B, D, F)**. Box and whisker plots show median (central line) 25th-75th percentile (box) and min to max (whiskers). Significance of Mann-Whitney unpaired non-parametric T-test are shown.
